# Systemic and cerebrospinal fluid biomarkers for tuberculous meningitis identification and treatment monitoring

**DOI:** 10.1128/spectrum.02246-23

**Published:** 2023-12-04

**Authors:** Xiang-Ping Yao, Jian-Chen Hong, Zai-Jie Jiang, Yu-Ying Pan, Xiao-Feng Liu, Jun-Mei Wang, Rui-Jie Fan, Bi-Hui Yang, Wei-Qing Zhang, Qi-Chao Fan, Li-Xiu Li, Bi-Wei Lin, Miao Zhao

**Affiliations:** 1 Department of Neurology, Institute of Neurology of First Affiliated Hospital, Institute of Neuroscience, and Fujian Key Laboratory of Molecular Neurology, Fujian Medical University, Fuzhou, China; 2 Department of Neurology, National Regional Medical Center, Binhai Campus of the First Affiliated Hospital, Fujian Medical University, Fuzhou, China; 3 Department of Gastrointestinal Surgery, The First Affiliated Hospital, Fujian Medical University, Fuzhou, China; 4 Department of Laboratory Medicine, The First Affiliated Hospital, Fujian Medical University, Fuzhou, China; 5 Department of Infectious Disease, The First Affiliated Hospital, Fujian Medical University, Fuzhou, China; 6 Department of Oncology, Fuzhou Pulmonary Hospital of Fujian, Fuzhou, China; University of Southern California, Duarte, California, USA

**Keywords:** tuberculous meningitis, cerebrospinal fluid, cytokines, biomarker, MIG

## Abstract

Tuberculous meningitis (TBM) is a severe infectious disease affecting the central nervous system, causing high mortality and disability. However, current diagnostic methods for TBM using cerebrospinal fluid (CSF) lack sensitivity and predictive biomarkers for prognosis. We conducted a study on cytokine profiles in CSF and serum samples from TBM patients to identify disease-specific biomarkers. Patients were categorized into three groups: TBM (*n* = 17), cryptococcal meningitis (*n* = 10), and non-infection (*n* = 6), and cytokine levels were quantified using a 48-plex panel. After treatment, we observed a significant reduction in the levels of 12 cytokines, indicating their potential use as biomarkers for treatment monitoring. Among them, monokine induced by interferon-γ (MIG) and interleukin-18 showed significant differences in serum or CSF cytokine levels compared to the control groups. CSF levels of MIG in TBM patients were negatively correlated with the CSF/blood glucose ratio (*r* = −0.4728, *P* = 0.0475). Positive correlations were found between CSF leukocyte counts and several cytokines, including fibroblast growth factor-basic, granulocyte colony-stimulating factor (G-CSF), monocyte chemotactic protein-3, macrophage inflammatory protein 1 alpha (MIP-1α), and tumor necrosis factor alpha. G-CSF and MIP-1α were also positively correlated with CSF protein levels. Receiver operating characteristic curve analysis revealed that MIG exhibited the highest area under the curve of 0.92 [95% confidence interval (CI) 0.82–1.00] with a sensitivity of 0.85 (95% CI 0.58–0.97) and a specificity of 0.87 (95% CI 0.62–0.98), making it a promising diagnostic biomarker for TBM. Our study provides valuable insights into TBM’s pathogenesis and identifies potential biomarkers for diagnosis and evaluating treatment monitoring.

**IMPORTANCE:**

Tuberculous meningitis is a life-threatening infection with high mortality and disability rates. Current diagnostic methods using cerebrospinal fluid (CSF) samples have limited sensitivity and lack predictive biomarkers for evaluating prognosis. This study’s findings reveal excessive activation of the immune response during tuberculous meningitis (TBM) infection. Notably, a strong negative correlation was observed between CSF levels of monokine induced by interferon-γ (MIG) and the CSF/blood glucose ratio in TBM patients. MIG also exhibited the highest area under the curve with high sensitivity and specificity. This study suggests that MIG may serve as a novel biomarker for differentiating TBM infection in CSF or serum, potentially leading to improved diagnostic accuracy and better patient outcomes.

## INTRODUCTION

Tuberculous meningitis (TBM) is an infectious disease of the central nervous system (CNS) caused by *Mycobacterium tuberculosis* (Mtb). It is the most severe form of Mtb infection, with a mortality rate ranging from 16% to 67% and a rate of disabling neurological sequelae of 32%, particularly in human immunodeficiency virus (HIV)-positive adults, despite adequate anti-tuberculosis therapy (1, 2). Early diagnosis of TBM is challenging due to the non-specific clinical symptoms and the lack of sensitive methods to accurately detect Mtb (3). Although efforts have been made to develop simplified tests for TBM, their diagnostic efficacy remains limited. For example, Xpert MTB/RIF, recommended by the World Health Organization for TB diagnosis, is considered one of the most advanced molecular biological technologies (4). However, its sensitivity for cerebrospinal fluid (CSF) samples is low, with a diagnostic rate of only 14.2% (5). Delayed diagnosis and treatment contribute to a poor prognosis (6). The improved technology of pathogen detection and the timely initiation of appropriate therapy are essential for the management of TBM. Therefore, there is an urgent need for new diagnostic tools to facilitate the timely identification of TBM.

Inflammatory protein biomarkers in the host may hold diagnostic value for TBM (7). The symptoms and sequelae of TBM are largely driven by the inflammatory response triggered by Mtb. It has been observed that excessive intracerebral inflammation contributes to the mortality rate of TBM, and corticosteroids, which act as broad anti-inflammatory drugs, are now administered adjunctively with anti-tubercular antibiotics to reduce the production of proinflammatory cytokines (2, 8). Consequently, the identification of specific cytokines as disease-specific biomarkers could have diagnostic significance and provide new insights into the pathophysiology of TBM (9 – 12). Cytokines, signaling molecules produced by various cells, regulate immune responses, inflammation, cell proliferation, and differentiation. They can have pro-inflammatory or anti-inflammatory effects, acting locally or systemically to coordinate immune responses and physiological processes by modulating interleukins, interferons, chemokines, and other mediators (13). Alterations in cytokine levels among TBM patients can impact immune system function and potentially influence the course of TBM in various ways (14). Compared to other types of meningitis, several cytokines such as interferon gamma (IFN-γ), tumor necrosis factor alpha (TNF-α), interleukin (IL)-1β, IL-6, and IL-10 are upregulated in the CSF of TBM patients (9, 11, 15). While the disease-specific host inflammatory response to TBM has been well documented, there are limited data describing the changes in cytokine concentrations in the CSF of TBM patients undergoing treatment.

In this study, we examined the expression of specific cytokines in the CSF and serum of patients with TBM. We also compared the cytokine levels between pre and posttreatment CSF samples from TBM patients. The objective of this study was to assess differences in the cytokine profiles among the TBM, cryptococcal meningitis (CM), and non-infection (NF) groups and to determine the predictive value of potential disease-specific biomarkers.

## RESULTS

### Clinical characteristics of enrolled patients

A total of 232 participants with suspected CNS infections were included in the analysis. Among them, CSF and serum samples from 17 patients with TBM and 16 non-TBM patients were analyzed. These patients were categorized into three groups based on their diagnosis: TBM (*n* = 17), CM (*n* = 10), and NF group (*n* = 6) (Tables 1 and 2; Fig. S1). The median age of patients in the TBM, CM, and NF groups was 51, 62, and 56 years, respectively. There were no significant differences in age and gender among the three groups (Table 2). In the TBM group, there were 10 patients with definite TBM, 2 patients with probable TBM, and 5 patients with possible TBM. All 17 patients in the TBM group showed positive responses to anti-TB treatment in terms of clinical and CSF manifestations. The CM cases were confirmed by the identification of *Cryptococcus neoformans* from CSF (*n* = 10). The NF group included patients with diagnoses such as Alzheimer’s disease, narcolepsy with cataplexy, cerebral infarction, transient ischemic attack, low intracranial pressure syndrome, and vascular parkinsonism (Table 1).

**TABLE 1:** Demographic data, CSF findings, diagnostic test results, and final diagnosis of participants^*a*^

No.	Sex	Age	SD	Main complaint	P (mmH_2_O)	WBC (/μL)	Glucose (mmol/L)	Protein (g/L)	AFB	qPCR	XPERT	IIS	CrAg	mNGS	Imaging feature	Diagnostic score	Responses to anti-TB	Final diagnosis
1	F	29	30	Fever, headache	360	70	1.87	1.81	−	+	−	−	−	−	Tuberculous granuloma of the right frontal lobe	Definite	+	Definite TBM
2	M	51	6	Dizziness, vomiting	300	396	1.7	2.28	−	−	−	−	−	Mtb	Enhancement in cerebellar tentorium and sulci	Definite	+	Definite TBM
3	F	48	3	Headache, fever	330	209	2.53	2.2	−	−	−	−	−	−	Enhancement in brain parenchyma and pia mater	10	+	Possible TBM
4	F	40	7	Weakness in both lower limbs	130	1011	2.4	3.21	−	−	−	−	−	−	Consider the possibility of demyelinating lesions	9	+	Possible TBM
5	M	58	9	Recurrent headache, fever	300	169	4	0.91	−	−	−	−	−	−	Bilateral frontal lobe ischemic foci	7	+	Possible TBM
6	F	72	3	Unresponsive	220	53	1.73	1.72	−	+	−	−	−	Mtb	Bilateral parietal and basal ganglia ischemic foci	Definite	+	Definite TBM
7	M	68	20	Headache	220	150	1.39	3.28	−	+	−	−	−	−	Multiple ischemic foci in white matter area	Definite	+	Definite TBM
8	M	43	21	Headache and fever	180	355	1.28	0.99	−	−	−	−	−	−	Lateral ventricular posterior horn empyema	8	+	Possible TBM
9	M	71	7	Headache and fever	50	19	2.79	1.53	−	−	−	−	−	−	Bilateral basal ganglia abnormal signal foci	12	+	Probable TBM
10	M	49	10	Fever, confusion	NA	343	3.23	4.05	−	+	−	−	−	−	Demyelinating changes in bilateral basal ganglia	Definite	+	Definite TBM
11	F	68	7	Headache, dizziness, fatigue	165	142	1.87	1.04	−	+	−	−	−	Mtb	Bilateral ischemic foci in multiple brain regions	Definite	+	Definite TBM
12	M	70	14	Recurrent limb convulsions, fever	60	176	2.11	5.17	−	−	−	−	−	−	Right frontal temporal parietal occipital lobe and insular meningoencephalitis	12	+	Probable TBM
13	M	38	240	Recurrent fever	210	102	2.24	1.88	−	+	−	−	−	Mtb	Enhancement of cerebellar tentorium meninges	Definite	+	Definite TBM
14	M	40	7	Headache, fever, confusion	330	116	2.68	1.8	−	−	+	−	−	Mtb	Enhancement of pia mater and ventricular wall	Definite	+	Definite TBM
15	F	57	60	Headache, fever	90	337	2.82	4.24	−	−	−	−	−	Mtb	Enhancement in brain parenchyma	Definite	+	Definite TBM
16	M	46	17	Headache, fever, confusion	NA	−	−	−	−	−	+	−	−	Mtb	NA	Definite	+	Definite TBM
17	M	53	8	Headache, fever	85	110	5.33	0.62	−	−	−	−	−	−	Enhancement in bilateral frontoparietal region	8	+	Possible TBM
18	M	63	60	Headache, confusion	120	26	3.95	0.54	−	−	−	−	−	C.n	Abnormal enhancement in multiple brain regions	NA	NA	CM
19	F	59	60	Fever, headache	260	37	2.69	0.73	−	−	−	+	−	C.n	Abnormal signals in multiple brain regions	NA	NA	CM
20	F	31	8	Headache	NA	229	2.57	0.6	−	−	−	−	+	−	No obvious abnormalities found	NA	NA	CM
21	F	72	10	Headache, fatigue	60	98	1.89	0.72	−	−	−	+	−	−	Abnormal enhancement of the meninges	NA	NA	CM
22	M	61	14	Fever, headache, unresponsiveness	160	110	2.16	2.08	−	−	−	−	−	C.n	Multiple abnormal signals in the brain with abnormal enhancement of the meninges	NA	NA	CM
23	M	73	30	Fever, cough and sputum	NA	10	2.95	0.46	−	−	−	−	+	−	Abnormal signal shadows in the left frontoparietal lobe and right basal ganglia area	NA	NA	CM
24	M	64	60	Headache, dizziness, diplopia	80	81	2.22	1.18	−	−	−	−	+	−	Enhancement in cerebellar meninges	NA	NA	CM
25	F	40	90	Headache, dizziness	280	21	1	0.65	−	−	−	+	−	C.n	Abnormal signals in bilateral frontal lobes	NA	NA	CM
26	M	65	30	Fever	80	225	3.92	0.93	−	−	−	+	−	C.n	Bilateral abnormal signal foci in basal ganglia	NA	NA	CM
27	M	27	10	Headache	330	292	2.08	0.8	−	−	−	+	−	C.n	Cerebellar tentorium enhancement	NA	NA	CM
28	F	58	730	Memory loss	NA	1	3.27	0.33	−	−	−	−	−	−	Multiple ischemic foci in bilateral frontal lobes	NA	NA	Alzheimer’s disease
29	M	70	1095	Excessive daytime sleepiness	NA	1	3.56	0.48	−	−	−	−	−	−	Cerebral atrophy	NA	NA	Narcolepsy with cataplexy
30	F	46	20	Right limb weakness	140	0	3.49	0.37	−	−	−	−	−	−	Right basal ganglia infarction	NA	NA	Cerebral infarction
31	F	54	30	Paroxysmal left limb weakness	70	1	3.33	0.34	−	−	−	−	−	−	Multiple ischemic foci in bilateral frontal and parietal lobes	NA	NA	Transient ischemic attack
32	F	39	7	Recurrent headache	80	3	3.06	0.32	−	−	−	−	−	−	No obvious abnormalities found	NA	NA	Low intracranial pressure
33	M	76	30	Difficulty walking	85	2	2.71	0.95	−	−	−	−	−	−	Brain atrophy and white matter degeneration	NA	NA	Vascular parkinsonism

^*a*^ No., case number; M, male; F, female; SD, symptom duration before admission (days); P, intracranial pressure; WBC, white blood cell; AFB, acid-fast bacilli; qPCR, quantitative polymerase chain reaction; XPERT, Xpert MTB/RIF; IIS, India ink stain; CrAg, cryptococcal antigen; mNGS, metagenomic next-generation sequence; NA, not available; −, negative; +, positive; Mtb, *Mycobacterium tuberculosis*; C.n, *Cryptococcus neoformans*.

**TABLE 2:** Clinical characteristics of the enrolled patients

	TBM	Controls		*P^a^*
		CM	NF	
*n*	17	10	6	–^*b*^
Age, median (range)	51 (29–72)	62 (27–73)	56 (39–76)	0.4381
Gender, male/female	11/6 (64.70%)	6/4 (60.00%)	2/4 (33.33%)	0.4905
Symptoms				
Fever, *n* (%)	12/17 (70.59%)	4/10 (40%)	0	–
Headache, *n* (%)	11/17 (64.71%)	8/10 (80%)	1/6 (16.67%)	–
Altered mental status, *n* (%)	4/17 (23.53%)	2/10 (20%)	0	–
CSF, median (range)				
Pressure, mmH_2_O	210 (50–360)	140 (60–330)	85 (70–140)	0.1345
White blood count, ×10^6^/L	160 (19–1011)	90 (10–292)	1 (0–3)	0.0015
Glucose, mmol/L	2.32 (1.28–5.33)	2.40 (1.00–3.95)	3.30 (2.71–3.56)	0.1356
Proteins, g/L	1.85 (0.62–5.17)	0.73 (0.46–2.08)	0.36 (0.32–0.95)	<0.0001

^*a*^ Compared between the TBM and control groups using the Mann-Whitney *U* test.

^*b*^ -, not available.

As shown in Table 2, most patients with TBM presented with fever (70.59%), headache (64.71%), and altered mental status (23.53%). Additionally, CSF findings, including white blood cell counts (160 × 10^6^/L vs 90 × 10^6^/L, 1 × 10^6^/L, *P* = 0.0015) and protein levels (1.85 g/L vs 0.73 g/L, 0.36 g/L, *P* < 0.0001), in patients with TBM were significantly different from the controls. The clinical characteristics of the enrolled patients are summarized in Table 2.

Table 3 presents the changes in clinical manifestations and CSF profiles after approximately 1 month of anti-TB therapy. All 11 patients showed a positive response to anti-TB treatment in terms of clinical manifestations. A significant difference in white blood cell counts (*P* = 0.0055), protein levels (*P* = 0.0336), and CSF/blood glucose ratio (*P* = 0.0350) in the CSF was observed after anti-TB treatment compared to the pretreatment conditions in these 11 TBM patients. Table S1 shows the details of the CSF profile change after 1 month of anti-tuberculous therapy.

**TABLE 3:** Main symptoms and biochemical changes in CSF in pretreated and posttreated TBM patients (*n* = 11)

	Pretreatment	Posttreatment	*P^a^*
Symptoms			
Fever, *n* (%)	8/11 (72.73%)	0/11 (0%)	–^*b*^
Headache, *n* (%)	4/11 (36.36%)	0/11 (0%)	–
Altered mental status, *n* (%)	3/11 (27.27%)	0/11 (0%)	–
CSF, median (range)			
Pressure, mmH_2_O	170 (60–300)	145 (130–280)	0.4792
White blood count, ×10^6^/L	109 (38–1011)	42 (5–104)	0.0055
Glucose, mmol/L	2.26 (1.7–4.55)	3.14 (1.69–7.13)	0.2164
CSF/blood glucose ratio (%)	29.91 (26.38–42.25)	33.87 (27.85–65.55)	0.0350
Proteins, g/L	2.20 (0.32–5.16)	0.77 (0.26–4.23)	0.0336

^*a*^ Compared between the pretreatment and posttreatment groups using the Mann-Whitney *U* test.

^*b*^ -, not available.

### Cytokine profiles in the CSF of enrolled patients

**FIG 1:**
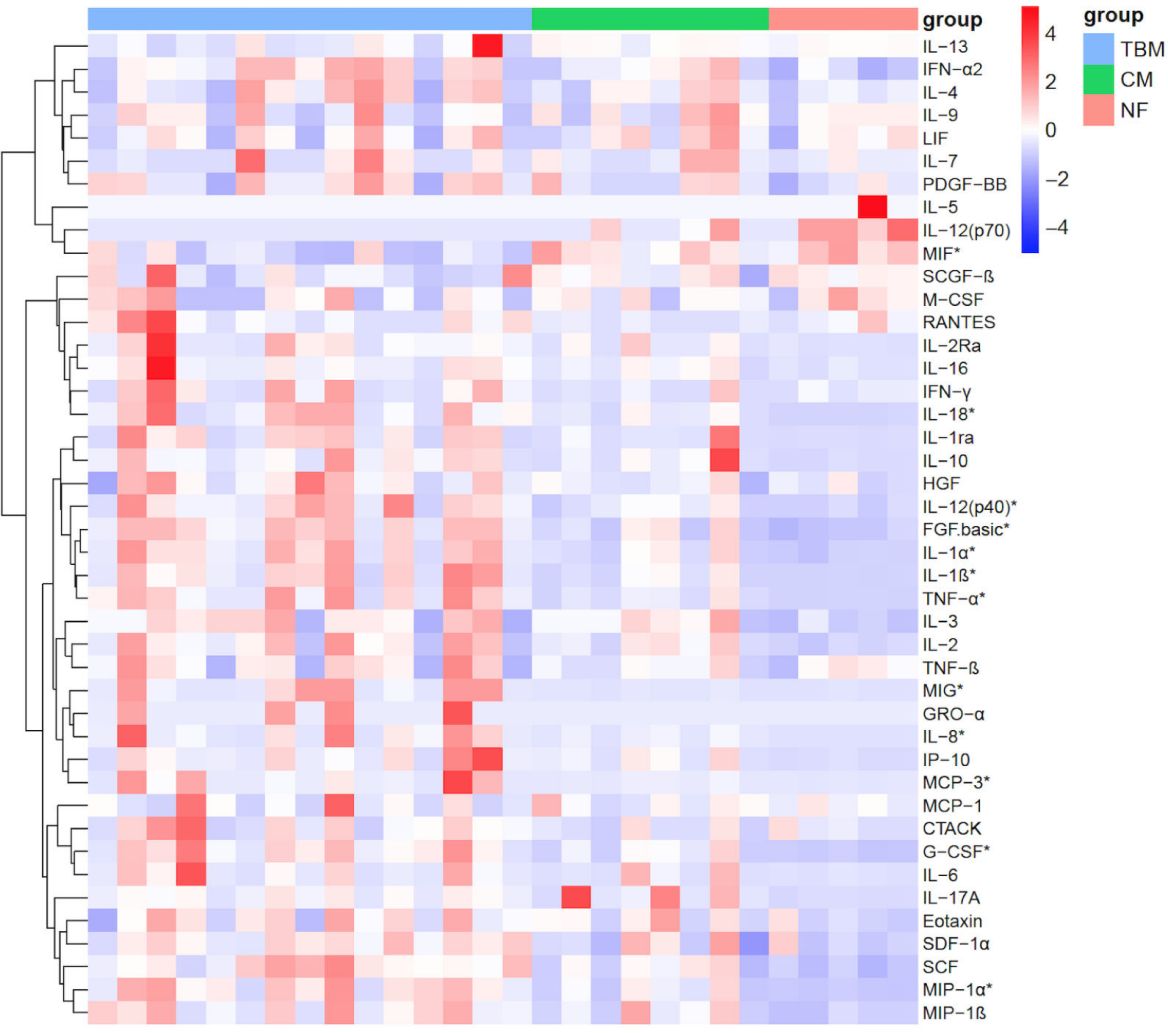
Heat map of cytokine profiles in the CSF of enrolled patients. TBM: *n* = 15, CM: *n* = 8, NF: *n* = 5. The levels of cytokines in CSF were measured by the Bio-Plex Pro Human Cytokine Screening 48-plex Panel. Significant cytokines between TBM and CM or NF (*P* < 0.05) were indicated with an asterisk. LIF, leukemia inhibitory factor; PDGF, platelet-derived growth factor; RANTES, regulated upon activation normal T cell expressed and secreted factor; HGF, hepatocyte growth factor; GRO, growth related gene; IP-10, IFN-γ inducible protein 10; CTACK, cutaneous T cell attracting chemokine; Eotaxin, eosinophil chemotactic protein; SDF, stromal cell derived factor.

**FIG 2:**
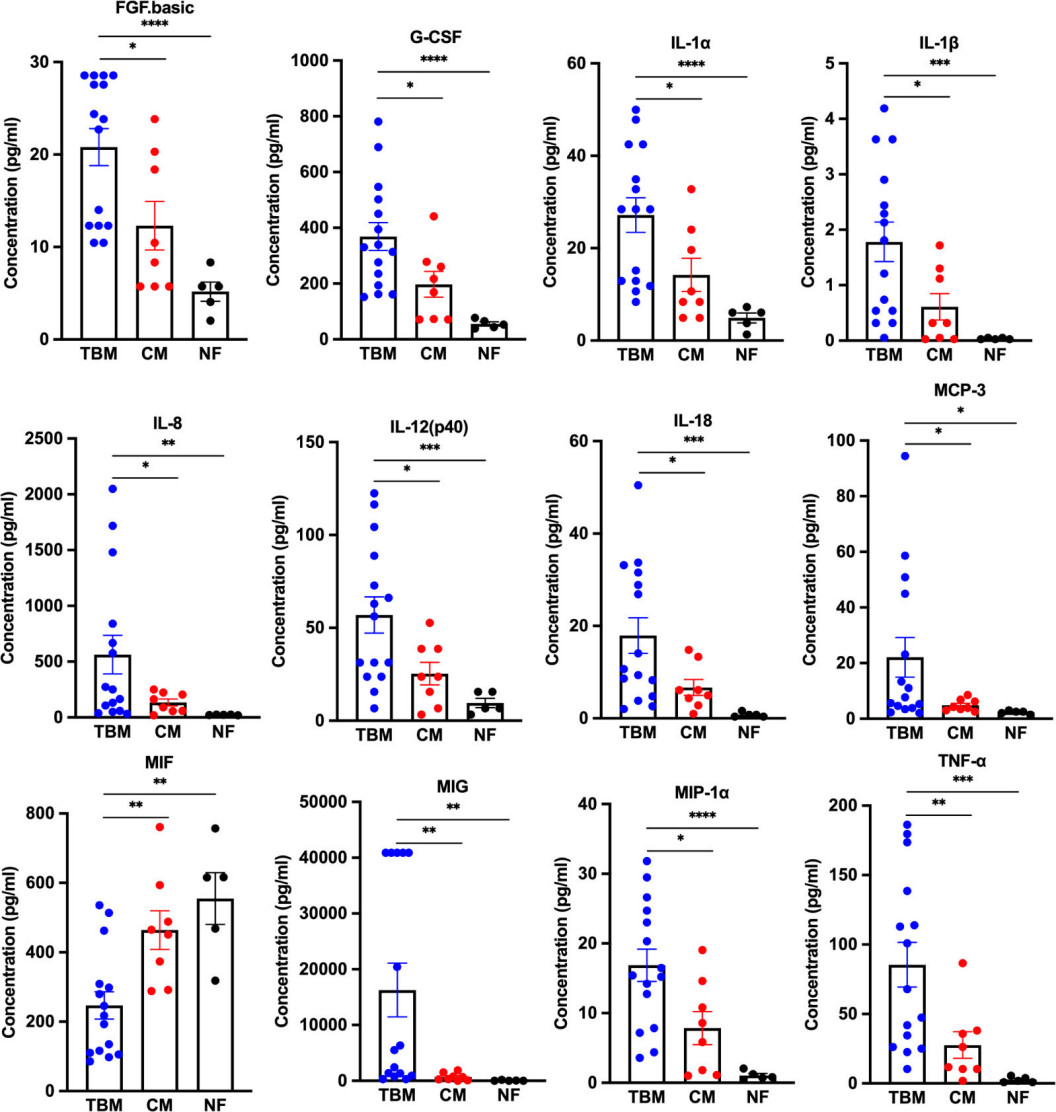
Concentration of significant cytokines between the TBM, CM, and NF. TBM: *n* = 15, CM: *n* = 8, NF: *n* = 5. The data in the graphs were reported as the mean ± SEM for each sample group. *P < 0.05, **P < 0.01, ***P < 0.001, ****P < 0.001. Different groups were compared using the Student’s *t*-test.

Among the 48 cytokines included in the panel, beta-nerve growth factor (β-NGF), granulocyte-macrophage colony-stimulating factor (GM-CSF), IL-15, TNF-related apoptosis-inducing ligand (TRAIL), and vascular endothelial growth factor (VEGF) were not detected in any of the groups and were therefore not considered in the present study. We observed that 25 out of 43 cytokines showed significantly elevated concentrations in the CSF of the TBM group compared to the NF group, including fibroblast growth factor-basic (FGF.basic), granulocyte colony-stimulating factor (G-CSF), IFN-α2, IFN-γ, IL-1α, IL-1β, IL-1rα, IL-2, IL-2Rα, IL-3, IL-6, IL-8, IL-10, IL-12(p70), IL-12(p40), IL-16, IL-17A, IL-18, IFN-γ inducible protein 10 (IP-10), monocyte chemotactic protein (MCP)-3, monokine induced by interferon-γ (MIG), macrophage inflammatory protein (MIP)-1α, MIP-1β, stem cell factor (SCF), and TNF-α (all *P* < 0.05). However, the level of macrophage migration inhibitor factor (MIF) was decreased in TBM as compared to the NF group (*P* = 0.0096) and the CM group (*P* = 0.0068) (Fig. 1 and 2; Table 3). Among these 25 elevated cytokines, 11 cytokines, including FGF.basic, G-CSF, IL-1α, IL-1β, IL-8, IL-12(p40), IL-18, MCP-3, MIG, MIP-1α, and TNF-α, also exhibited similar trends in the TBM group when compared to the CM group (all *P* < 0.05) (Fig. 1 and 2; Table 4). Cluster analyses revealed three cytokine clusters in TBM patients, in which the cytokine levels in each cluster showed strong positive correlations (Fig. S2). In addition, strong positive correlations with each other among the 11 cytokines were observed.

**TABLE 4:** Concentration of cytokines (pg/mL) in CSF between the TBM and control groups^*a*^

Cytokines	TBM (*n* = 15)	CM (*N* = 8)	NF (*N* = 5)	TBM vs CM *P*-value	TBM vs NF *P*-value
CTACK	8.58 (3.59, 15.45)	2.81 (2.37, 6.38)	4.37 (2.81, 5.83)	0.064528024	0.133949572
Eotaxin	3.32 (2.36, 4.71)	3.44 (1.94, 3.84)	1.87 (1.73, 2.3)	0.750865068	0.094527353
FGF.basic	23.84 (12.32, 28.05)	9.39 (5.73, 18.86)	5.73 (4.07, 5.73)	0.02076884	1.69496E−06
G-CSF	330.64 (214.51, 476.28)	192.62 (72.43, 264.52)	52.74 (43.34, 65.13)	0.020521672	1.9419E−05
GRO-α	201 (201, 394.55)	201 (201, 201)	201 (201, 201)	0.047719292	0.047719292
HGF	339.58 (263.05, 491.53)	271.36 (232.3, 296.96)	236.99 (218.16, 277.81)	0.085192467	0.065298321
IFN-α2	9.27 (6.94, 13.08)	7.56 (6.43, 9.77)	4.02 (1.87, 5.61)	0.310056063	0.005479043
IFN-γ	1.3 (0.29, 5.19)	0.19 (0.17, 0.75)	0.94 (0.38, 0.94)	0.066803483	0.025895498
IL-1α	28.41 (12.93, 38.7)	9.53 (7.52, 20.73)	6.09 (3.75, 6.09)	0.021592153	3.03444E−05
IL-1β	1.81 (0.54, 2.67)	0.32 (0.04, 1.17)	0.03 (0.03, 0.05)	0.012051337	0.000224336
IL-1rα	2,460.06 (493.26, 3,559.16)	670.45 (439.92, 891.18)	386.46 (362.83, 408.79)	0.361534363	0.001079274
IL-2	3.23 (1.72, 5.5)	2.23 (1.54, 3.8)	0.65 (0.65, 1.01)	0.354107223	0.001036478
IL-2Rα	29.58 (15.27, 64.39)	9.4 (0.35, 50.14)	4.04 (0.4, 4.81)	0.230259896	0.014753851
IL-3	1.54 (1.16, 1.91)	1.25 (1.16, 1.63)	0.46 (0.36, 0.57)	0.928416389	0.001404594
IL-4	3.27 (1.72, 4.58)	2.81 (2.33, 3.54)	2.35 (2.05, 2.49)	0.760954058	0.077452217
IL-5	3.22 (3.22, 3.22)	3.22 (3.22, 3.22)	3.22 (3.22, 3.22)	1	0.373900966
IL-6	9.99 (4.64, 43.94)	1.97 (0.16, 31.48)	0.64 (0.16, 1.15)	0.539314186	0.014231899
IL-7	1.57 (1.57, 7.01)	3.14 (2.75, 8.47)	3.14 (3.14, 3.14)	0.799018434	0.425154142
IL-8	250.45 (83.98, 754)	127.11 (58.02, 209.81)	24.58 (21.68, 25.68)	0.027553009	0.007534987
IL-9	8.33 (3.44, 13.35)	13.33 (6.04, 16.43)	13.35 (11.73, 13.35)	0.492408367	0.860837973
IL-10	20.42 (17.62, 44.6)	16.81 (6.92, 26.22)	3.69 (2.76, 4.6)	0.952792838	0.000609713
IL-12(p70)	0.15 (0.15, 0.15)	0.15 (0.15, 0.38)	0.9 (0.6, 0.9)	0.135272637	0.027769613
IL-12(p40)	56.15 (27.48, 80.76)	23.66 (13.34, 38.66)	6.64 (6.64, 15.57)	0.012004063	0.000248394
IL-13	3.84 (2.62, 6.2)	6.97 (6.48, 7.29)	6.66 (6.66, 6.84)	0.885432849	0.93988459
IL-16	19.79 (12.79, 30.45)	15.2 (8.99, 22.81)	7.65 (7.65, 8.99)	0.215888098	0.024351306
IL-17A	5.03 (3.19, 6.63)	2.72 (1.8, 17.77)	0.39 (0.39, 0.86)	0.291009485	6.14199E−06
IL-18	10.7 (6.52, 30.22)	5.56 (3.99, 7.93)	0.76 (0.38, 0.76)	0.01544104	0.000538923
IP-10	2,336.34 (1,903.53, 7,156.12)	2,424.5 (1,075.81, 4,448.24)	610.17 (594.31, 650.05)	0.23684296	0.00638351
LIF	27.96 (11.44, 35.19)	27.85 (16.67, 42.07)	27.96 (25.44, 32.84)	0.516620074	0.98506084
M-CSF	5.01 (0.22, 7.44)	5.31 (3.74, 5.79)	6.79 (5.9, 7.37)	0.968117326	0.462357449
MCP-1	67.67 (28.47, 149.41)	140.28 (64.8, 174.13)	100.03 (87.63, 145.97)	0.944801987	0.68086667
MCP-3	7.69 (4.22, 34.05)	4.04 (3.34, 6.39)	2.57 (2.06, 2.57)	0.030038337	0.015010575
MIF	217.35 (113.38, 303.86)	458.14 (353.2, 514.57)	615.64 (468.34, 616.56)	0.006802024	0.00960422
MIG	5,528.89 (1,146.89, 40,890.62)	491.55 (233.32, 969.7)	40.25 (32.44, 45.14)	0.006008038	0.00461829
MIP-1α	15.41 (10.3, 23.87)	7.22 (1.62, 11.76)	0.83 (0.73, 1.26)	0.013676543	7.66521E−06
MIP-1β	31.97 (21.88, 47.54)	23.23 (13.02, 32.96)	11.92 (6.92, 11.92)	0.279770729	4.45856E−05
PDGF-BB	37.59 (22.16, 41.44)	22.16 (18.29, 39.99)	22.16 (18.29, 22.16)	0.602417476	0.103415699
RANTES	7.02 (0.09, 16.3)	1.69 (0.16, 3.71)	5.56 (4.01, 7.02)	0.030408361	0.520942886
SCF	40.76 (38.09, 56.72)	37.05 (22.19, 41.47)	20.56 (16.17, 24.02)	0.050770434	7.33608E−06
SCGF-β	14,892.75 (13,503.2, 20,393.49)	19,797.67 (15,874.58, 21,847.12)	21,218.69 (20,752.43, 21,686.09)	0.934672769	0.359385412
SDF-1α	602.8 (496.76, 719.5)	399.62 (305.66, 672.54)	365.67 (311.5, 397.41)	0.273268772	0.089776698
TNF-α	67.89 (30.41, 126.26)	19.02 (10.34, 36.31)	1.98 (0.99, 3.9)	0.005721582	0.000149063
TNF-β	13.6 (5.32, 16.59)	10.1 (6.48, 10.54)	11.86 (10.54, 14.03)	0.316607248	0.629053228

^*a*^ The data were presented as the median (interquartile range), and two groups were compared using the Student’s *t*-test. LIF, leukemia inhibitory factor; PDGF, platelet-derived growth factor; SCGF, stem cell growth factor; M-CSF, macrophage colony-stimulating factor; RANTES, regulated upon activation normal T cell expressed and secreted factor; HGF, hepatocyte growth factor; GRO, growth related gene; CTACK, cutaneous T cell attracting chemokine; Eotaxin, eosinophil chemotactic protein; SDF, stromal cell derived factor.

### Dynamics of cytokines after 1 month of anti-tuberculosis treatment

**FIG 3:**
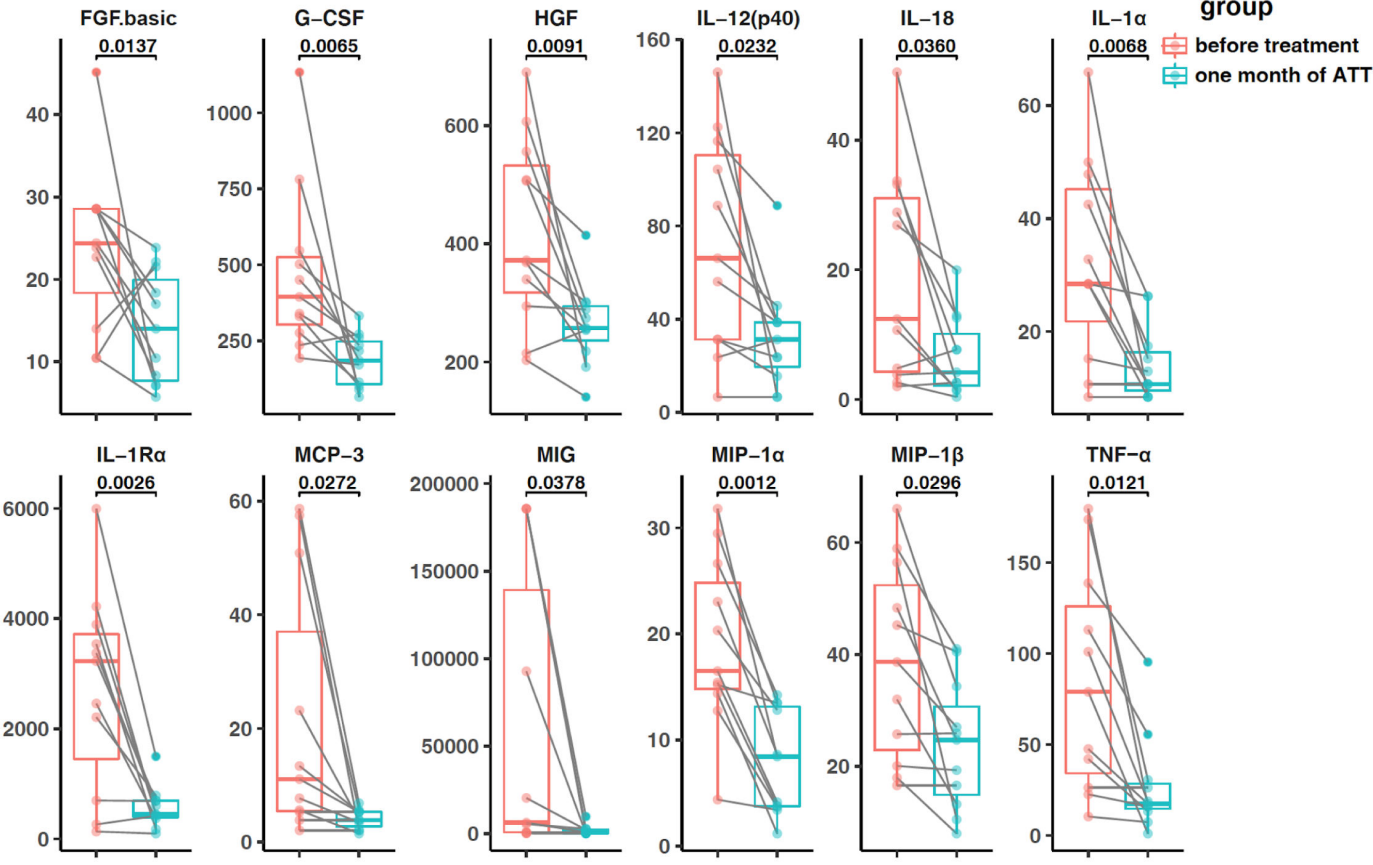
Concentration of significant cytokines in the TBM group after 1 month of therapy. The boxplots were used to display the trend before and after the treatment, and the lines represented the trend of the same individual before and after the treatment. The groups were compared using the Student’s *t*-test.

Cytokine estimation was performed on posttreatment CSF samples obtained from 11 TBM patients. After approximately 1 month of therapy, all 11 patients showed improved clinical symptoms and CSF findings (Table 3). The concentration of CSF cytokines in the TBM group generally decreased following treatment. Specifically, 12 out of the 43 detected cytokines showed significant differences, including FGF.basic, G-CSF, hepatocyte growth factor (HGF), IL-1α, IL-1rα, IL-12(p40), IL-18, MCP-3, MIG, MIP-1α, MIP-1β, and TNF-α (Fig. 3). Among these decreased cytokines, the levels of nine cytokines (FGF.basic, G-CSF, IL-1α, IL-12(p40), IL-18, MCP-3, MIG, MIP-1α, and TNF-α) remained significantly higher in the TBM group than in the control groups (Table 4).

### Cytokine profiles in the serum of enrolled patients

**FIG 4:**
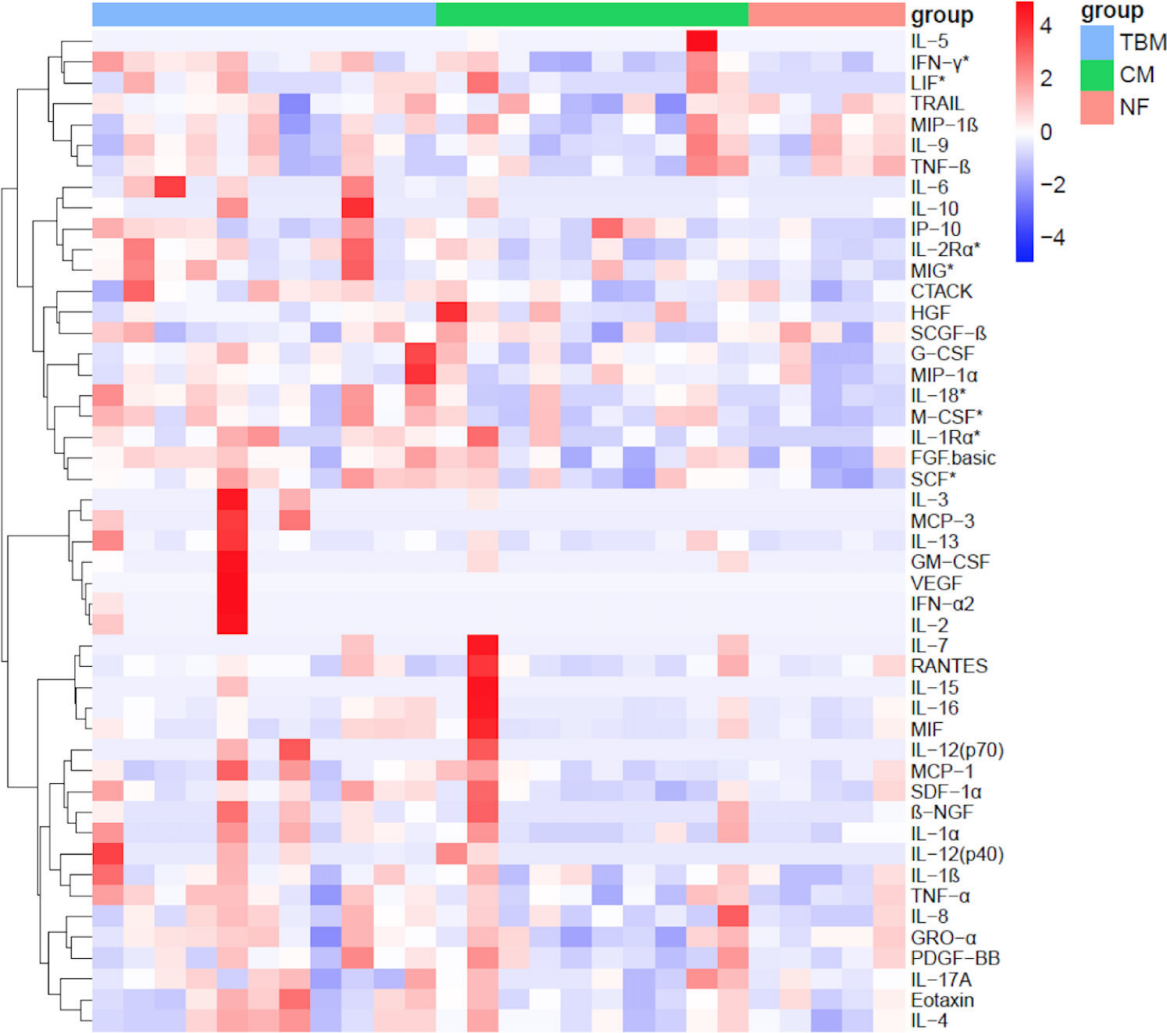
Heat map of cytokine profiles in the serum of enrolled patients. TBM: *n* = 11, CM: *n* = 10, NF: *n* = 5. The levels of cytokines in serum were measured by the Bio-Plex Pro Human Cytokine Screening 48-plex Panel. Significant cytokines between TBM and NF (*P* < 0.05) were indicated with an asterisk. PDGF, platelet-derived growth factor; SCGF, stem cell growth factor; RANTES, regulated upon activation normal T cell expressed and secreted factor; GRO, growth related gene; IP-10, IFN-γ inducible protein 10; CTACK, cutaneous T cell attracting chemokine; Eotaxin, eosinophil chemotactic protein; SDF, stromal cell derived factor.

We also measured the levels of 48 cytokines in the serum of the 3 groups. Among the 48 cytokines, we observed significantly higher concentrations of serum IFN-γ, IL-1rα, IL-2Rα, IL-18, leukemia inhibitory factor (LIF), macrophage colony-stimulating factor (M-CSF), MIG, and SCF in the TBM group than in the NF group (Fig. 4; Table 5). Notably, MIG and IL-18 were also remarkably elevated in the CSF of the TBM group compared to the control groups and met the statistical difference in the reduction of TBM CSF after treatment. In addition, only IL-18 was significantly higher in the TBM serum than in the CM group.

**TABLE 5:** Concentration of cytokines in serum between the TBM and control groups^*a*^

Cytokines	TBM (*N* = 11)	CM (*N* = 10)	NF (*N* = 5)	TBM vs CM *P-*value	TBM vs NF *P-*value
β-NGF	2.37 (2.37, 7.78)	2.37 (2.37, 2.37)	2.37 (2.37, 2.37)	0.823487491	0.096762049
CTACK	878.07 (695.58, 955.6)	757.57 (671.65, 862.88)	687.41 (584.07, 753.45)	0.230929196	0.227909493
Eotaxin	74.6 (42.42, 90.67)	58.37 (45.48, 64.43)	49.27 (47.88, 70.99)	0.367768165	0.335392845
FGF.basic	37.75 (33.33, 43.74)	30.9 (22.94, 44.69)	6.5 (6.5, 33.33)	0.257284401	0.069358995
G-CSF	83.59 (73.72, 101.53)	81.94 (67.08, 96.66)	70.41 (29.81, 70.41)	0.334066068	0.14210579
GM-CSF	0.85 (0.85, 0.85)	0.85 (0.85, 0.85)	0.85 (0.85, 0.85)	0.54966393	0.311440301
GRO-α	1,998.8 (1,715.7, 2,294.64)	1,393.2 (1,211.01, 2,314.79)	1,898.87 (1,658.9, 1,898.87)	0.375687882	0.812180532
HGF	578.57 (506.33, 680.4)	528.78 (358.33, 1,292.4)	433.21 (333.02, 482.23)	0.237024615	0.102199471
IFN-α2	3.74 (3.74, 3.74)	3.74 (3.74, 3.74)	3.74 (3.74, 3.74)	0.278556372	0.278556372
IFN-γ	10.17 (6.65, 12.25)	6.29 (2.65, 10.17)	5.22 (4.49, 5.22)	0.187180227	0.002471011
IL-1α	10.22 (5.35, 33.53)	5.35 (0.15, 22.02)	5.35 (5.35, 14.99)	0.511354943	0.092725041
IL-1β	2.15 (2.15, 3.72)	2.57 (1.49, 3.55)	1.27 (0.19, 2.97)	0.541029912	0.186956782
IL-1rα	540.82 (272.74, 704.2)	272.74 (93.67, 395.6)	74.94 (74.94, 74.94)	0.517637612	0.008716354
IL-2	0.54 (0.54, 0.54)	0.54 (0.54, 0.54)	0.54 (0.54, 0.54)	0.239067703	0.239067703
IL-2Rα	70.08 (60.43, 100.33)	50.58 (29.43, 80.57)	44.21 (36.3, 54.34)	0.065798515	0.017573112
IL-3	0.28 (0.28, 0.28)	0.28 (0.28, 0.28)	0.28 (0.28, 0.28)	0.270491145	0.214062424
IL-4	8.81 (4.28, 9.66)	6.3 (5.23, 6.97)	5.61 (4.08, 6.3)	0.487825112	0.099255669
IL-5	16.5 (16.5, 16.5)	16.5 (16.5, 16.5)	16.5 (16.5, 16.5)	0.310232397	1
IL-6	0.66 (0.66, 9.18)	0.66 (0.66, 0.66)	0.66 (0.66, 0.66)	0.091598586	0.061030008
IL-7	6.28 (6.28, 6.28)	6.28 (6.28, 6.28)	6.28 (6.28, 6.28)	0.347931918	0.340893132
IL-8	9.83 (3.43, 13.84)	5.4 (0.44, 10.6)	2.1 (0.35, 3.45)	0.721224745	0.136153312
IL-9	1,154.68 (985.88, 1,274.64)	1,090.74 (1,028.95, 1,179.53)	1,190.34 (1,039.82, 1,254.05)	0.997260305	0.728554772
IL-10	1.59 (1.59, 2.39)	1.59 (1.59, 1.59)	1.59 (1.59, 1.59)	0.322514327	0.218129664
IL-12(p70)	0 (0, 0)	0 (0, 0)	0 (0, 0)	0.797390272	0.192127031
IL-12(p40)	2.62 (2.62, 15.91)	2.62 (2.62, 2.62)	2.62 (2.62, 2.62)	0.566291072	0.126707269
IL-13	2.2 (1.63, 3.24)	1.63 (1.06, 2.98)	1.06 (1.06, 1.06)	0.273752376	0.086748919
IL-15	41.74 (41.74, 41.74)	41.74 (41.74, 41.74)	41.74 (41.74, 41.74)	0.492830468	0.340893132
IL-16	51.17 (46.22, 92.37)	41.15 (39.22, 51.17)	35.96 (30.6, 46.22)	0.627242467	0.1397022
IL-17A	9.04 (5.32, 12.75)	8.12 (7.19, 13.43)	8.12 (7.19, 9.04)	0.757627632	0.743608533
IL-18	68.1 (57.74, 118.99)	33.7 (21.67, 63.49)	23.02 (22.25, 26.11)	0.049093184	0.002483157
IP-10	923.19 (330.62, 996.02)	492.92 (401.06, 760.49)	270.03 (269.04, 423.53)	0.77394625	0.06351593
LIF	17.6 (8.8, 45.75)	13.2 (8.8, 38.71)	8.8 (8.8, 8.8)	0.914020843	0.013810906
M-CSF	32.93 (28.31, 49.74)	24.79 (20.03, 44.07)	16.98 (13.25, 20.03)	0.241392808	0.002514167
MCP-1	14.06 (8.28, 23.97)	12.96 (9.83, 21.13)	14.06 (14.06, 16.17)	0.738796443	0.515895269
MCP-3	0.38 (0.38, 1.7)	0.38 (0.38, 0.38)	0.38 (0.38, 0.38)	0.109889728	0.109889728
MIF	530.26 (224.36, 878.63)	272.38 (199.75, 444.73)	273.27 (192.98, 396.6)	0.777166842	0.198822971
MIG	442.54 (267.82, 948.86)	333 (258.99, 532.32)	177.37 (99.33, 327.03)	0.257305992	0.028676062
MIP-1α	3.25 (2.75, 3.71)	2.88 (2.75, 3.68)	2.48 (1.75, 3.25)	0.498465019	0.287674712
MIP-1β	258.4 (240.52, 284.24)	254.98 (238.4, 276.04)	267.3 (264.35, 285.33)	0.845943509	0.276725244
PDGF-BB	1,894.05 (1,135.73, 2,577.5)	1,349.51 (944.7, 2,626.94)	1,479.61 (1,433.67, 1,602.23)	0.899575804	0.484457751
RANTES	11,644.96 (9,803.65, 13,307.92)	8,546.15 (6,798.2, 12,731.4)	10,615.26 (8,947.76, 11,170.79)	0.643729525	0.949125686
SCF	94.36 (89.19, 118.19)	92.64 (69.98, 112.28)	68.22 (53.92, 82.25)	0.218595712	0.008756915
SCGF-β	56,619.37 (51,240.71, 69,966.8)	62,870.68 (46,578.32, 67,895.01)	63,668.4 (63,440.41, 65,836.55)	0.895583523	0.73944093
SDF-1α	1,110.15 (876.78, 1,157.27)	774.65 (693.8, 935.59)	848.53 (752.53, 869.62)	0.241312365	0.146494764
TNF-α	61.23 (52.9, 73.3)	52.9 (42.81, 65.92)	44.3 (41.35, 47.2)	0.258491182	0.112590226
TNF-β	884.18 (797.58, 1,002.99)	866.72 (796.85, 994.25)	997.87 (866.71, 1,076.78)	0.669083836	0.280316353
TRAIL	31.49 (27.66, 36.72)	29.97 (16.06, 37.09)	34.49 (26.89, 41.83)	0.552170751	0.601714912
VEGF	48 (48, 48)	48 (48, 48)	48 (48, 48)	0.340893132	0.340893132

^*a*^ The data were presented as median (interquartile range), and two groups were compared using the Student’s *t*-test. PDGF: platelet-derived growth factor; SCGF: stem cell growth factor; RANTES: regulated upon activation normal T cell expressed and secreted factor; GRO: growth related gene, CTACK: cutaneous T cell attracting chemokine; Eotaxin, eosinophil chemotactic protein; SDF: stromal cell derived factor.

### Correlations between CSF cytokine levels and CSF parameters in TBM patients

**FIG 5:**
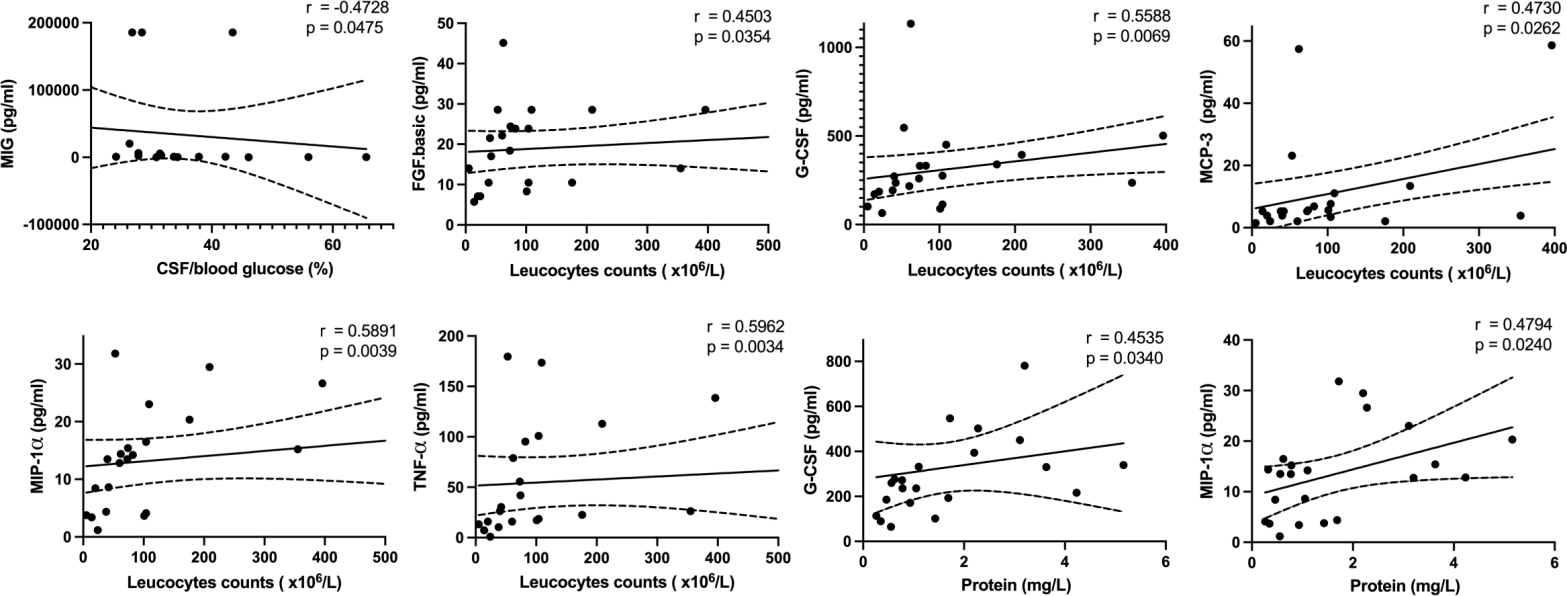
The correlation between CSF parameters and CSF cytokine levels in patients with TBM. The CSF/blood glucose ratio was negatively correlated with CSF MIG levels in the TBM group (*r* = −0.4728, *P* = 0.0475). CSF leukocyte counts (×10^6^/L) were positively correlated with CSF FGF.basic (*r* = 0.4503, *P* = 0.0354), G-CSF (*r* = 0.5588, *P* = 0.0069), MCP-3 (*r* = 0.4730, *P* = 0.0262), MIP-1α (*r* = 0.5891, *P* = 0.0039) and TNF-α (*r* = 0.5962, *P* = 0.0034). CSF protein (mg/L) was positively correlated with G-CSF (*r* = 0.4535, *P* = 0.0340) and MIP-1α (*r* = 0.4794, *P* = 0.0240).

Principal component analysis (PCA) on the cytokines demonstrated that clear separations of the groups (TBM, CM, and NF) were observed in CSF, serum, and 1 month after anti-tuberculosis treatment (ATT) (Fig. S3). Among all the CSF cytokines, the nine cytokines [FGF.basic, G-CSF, IL-1α, IL-12(p40), IL-18, MCP-3, MIG, MIP-1α, and TNF-α] exhibited the most distinct differences between the TBM and control groups. These nine cytokines also showed significant decreases after treatment. Notably, MIG and IL-18 also showed the most significant differences in serum compared to the NF group. To determine the correlation between the levels of the nine CSF cytokines and CSF parameters, we performed a correlation analysis. We found a strong negative correlation between CSF/blood glucose ratio and CSF MIG level in patients with TBM (*r* = −0.4728, *P* = 0.0475). Positive correlation was seen in CSF leukocyte counts (×10^6^/L) with FGF.basic (*r* = 0.4503, *P* = 0.0354), G-CSF (*r* = 0.5588, *P* = 0.0069), MCP-3 (*r* = 0.4730, *P* = 0.0262), MIP-1α (*r* = 0.5891, *P* = 0.0039), and TNF-α (*r* = 0.5962, *P* = 0.0034). Among these cytokines, G-CSF (*r* = 0.4535, *P* = 0.0340) and MIP-1α (*r* = 0.4794, *P* = 0.0240) were also positively correlated with CSF protein levels (Fig. 5).

### Potential biomarkers of TBM

**FIG 6:**
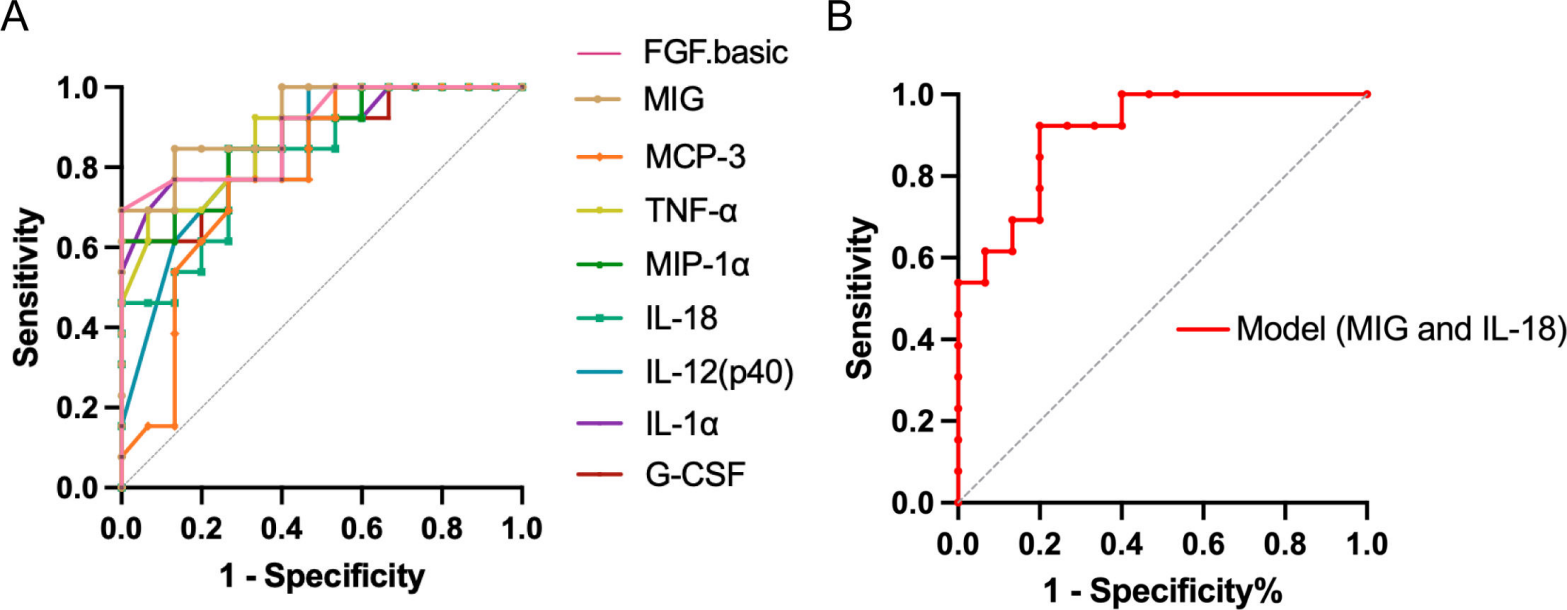
ROC curves. ROC curves of significant cytokines (**A**) and the model (**B**) in the prediction of TBM.

We performed receiver operating characteristic (ROC) curve analysis to evaluate whether the aforementioned nine cytokines could serve as potential CSF biomarkers for TBM. The results indicated that all of them had an area under the curve (AUC) ≥0.75, strongly suggesting their potential as CSF biomarkers for TBM (Fig. 6A; Table 6). Among them, MIG exhibited the highest predictive efficiency [AUC: 0.92, 95% confidence interval (CI) 0.82–1.00] with a sensitivity of 0.85 (95% CI 0.58–0.97) and a specificity of 0.87 (95% CI 0.62–0.98) when a cutoff value of 783.30 pg/mL was used. Furthermore, we constructed a prediction model for TBM using MIG and IL-18 and evaluated its efficiency. The prediction model achieved a higher sensitivity of 0.92 (95% CI 0.67–1.00) with a high predictive efficiency (AUC: 0.91, 95% CI 0.80–1.00) and specificity of 0.80 (95% CI 0.55–0.93) (Fig. 6B; Table 6).

**TABLE 6:** Predictive performance indices of significantly changed cytokines in the prediction of TBM

Cytokines	*P*-value	AUC (95% CI)	Cutoff value	Sensitivity (95% CI)	Specificity (95% CI)
FGF.basic	0.0004	0.89 (0.78–1.00)	<9.40	0.69 (0.42–0.87)	1.00 (0.80–1.00)
G-CSF	0.0014	0.86 (0.72–1.00)	<114.90	0.62 (0.36–0.82)	1.00 (0.80–1.00)
IL-1α	0.0007	0.88 (0.74–1.00)	<11.24	0.77 (0.50–0.92)	0.87 (0.62–0.98)
IL-12(p40)	0.0030	0.83 (0.68–0.98)	<54.44	1.00 (0.77–1.00)	0.53 (0.30–0.75)
IL-18	0.0034	0.83 (0.67–0.98)	<7.20	0.85 (0.58–0.97)	0.73 (0.48–0.89)
MCP-3	0.0113	0.78 (0.61–0.96)	<4.42	0.77 (0.50–0.92)	0.73 (0.48–0.89)
MIG	0.0002	0.92 (0.82–1.00)	<783.30	0.85 (0.58–0.97)	0.87 (0.62–0.98)
MIP-1α	0.0008	0.87 (0.74–1.00)	<2.83	0.62 (0.36–0.82)	1.00 (0.80–1.00)
TNF-α	0.0007	0.88 (0.76–1.00)	<17.16	0.69 (0.42–0.87)	0.93 (0.70–1.00)
Model	0.0003	0.91 (0.80–1.00)	–^*b*^	0.92 (0.67–1.00)	0.80 (0.55–0.93)

^*a*^ AUC, area under curve; FGF.Basic, fibroblast growth factor-basic; G-CSF, granulocyte colony-stimulating factor; IL, interleukin; MCP-3: monocyte chemotactic protein 3; MIG: monokine induced by IFNγ; MIP-1α, macrophage inflammatory protein-1 alpha; TNF-α: tumor necrosis factor alpha.

^*b*^ -, not available.

### MIG levels in the CSF and serum of TBM patients and controls by enzyme-linked immunosorbent assay

To further confirm the usefulness of MIG as a biomarker to identify TBM, a classic enzyme-linked immunosorbent assay (ELISA) kit was used to detect the MIG concentrations in both the CSF and serum of TBM patients and controls. The results showed that MIG concentration in CSF was significantly higher than that in the controls (CM group, *P* = 0.0030 and NF group, *P* = 0.0021) (Table S2). It was noteworthy that mean CSF MIG was approximately 100-fold higher in TBM than in NF. The serum MIG levels of TBM were also significantly higher than those of the NF group (*P* = 0.0202), but not statistically significant as compared to those of the CM group (*P* = 0.0843), which was consistent with the results conducted by the Luminex assay (Table S2; Table 5).

## DISCUSSION

TBM is a severe complication of tuberculosis that often leads to high mortality or disability, and the diagnosis of TBM is challenging. The clinical presentation of TBM, including symptoms and complications, is primarily influenced by the host’s inflammatory response to the infection (14). Mtb infection triggers the production of various pro-inflammatory cytokines, such as IFN-γ, IL-6, IL-1β, and TNF-α (15, 16). These cytokines contribute to inflammation in the brain, resulting in meningeal irritation, vasculitis, and increased permeability of the blood-brain barrier. Additionally, cytokines attract immune cells, including macrophages and neutrophils, to the infection site, contributing to the formation of granulomas, a characteristic feature of tuberculosis (15). In TBM, anti-inflammatory cytokines such as IL-10 and TGF-β play a critical role in regulating the immune response, preventing excessive inflammation, and modulating the differentiation and activation of immune cells to ensure an appropriate immune response to the infection. Previous studies have suggested that multiple cytokines in the CSF are elevated to varying degrees in different etiologies of meningitis, indicating the potential for identifying biomarkers for differential diagnosis (10, 17). Moreover, there is limited information available on the correlation of CSF cytokines in TBM patients undergoing treatment. Therefore, the identification of disease-specific biomarkers may hold diagnostic and therapeutic value and enhance our understanding of the pathogenesis of TBM.

In order to gain further insights into the neuro-inflammatory processes related to cytokine profiles and identify potential biomarkers, we conducted a comprehensive evaluation of cytokine profiles in TBM patients. We investigated a panel of 48 biomarkers in both their CSF and serum and assessed their changes during treatment in conjunction with standard CSF parameters. Compared to the non-infection group, we observed elevated levels of the majority of measured cytokines (25/48) in the CSF during Mtb infection, including TNF-α, IFN-γ, IL-1β, IL-2, IL-3, IL-6, IL-8, IL-10, IL-12(p40), IL-17A, IL-18, IP-10, MIG, MCP-3, MIP-1α, and MIP-1β. These findings are consistent with previous studies (11, 15, 16, 18). The concentrations of T helper type 1 (Th1) cytokines [such as TNF-α, IL-1β, IL-2, IL-12(p40), IL-18, and IFN-γ], representative Th2-type cytokines (such as IL-10), and Th17-type cytokines (such as IL-17A) were all implicated in TBM. When comparing TBM patients with CM patients, we found that 12 of these cytokines exhibited a similar trend in TBM. Notably, the levels of TNF-α were significantly higher in TBM patients, indicating a potential protective role against Mtb through granulomata, which is consistent with previous studies (15, 18, 19). In mouse models, the absence of the IL-12p40 subunit increased susceptibility to mycobacteria, and administration of exogenous or endogenous IL-12p40 decreased the bacterial burden (20).

We proceeded to evaluate the changes in cytokine levels among the 11 patients after 1 month of anti-tuberculous therapy, encompassing the 48 cytokines previously assessed. Our findings revealed significant reductions in the levels of FGF.basic, G-CSF, HGF, IL-1α, IL-1rα, IL-12(p40), IL-18, MCP-3, MIG, MIP-1α, MIP-1β, and TNF-α in the CSF samples. These results were consistent with expectations for an inflammatory process, indicating the significant involvement of these cytokines. Previous studies have also demonstrated the protective role of decreased levels of IL-1α, MIP-1α, MIG, and TNF-α in the pathogenesis of TBM, which aligns with the findings of our current research (9, 21 – 23). Notably, we observed remarkably higher levels of FGF.basic, G-CSF, IL-1α, IL-12(p40), IL-18, MCP-3, MIG, MIP-1α, and TNF-α in the TBM group. Specifically, we identified that the actual concentrations of MIG were 100-fold higher than those in the NF group, nearly 30-fold higher than the levels after treatment, and significantly elevated in the serum of the TBM group. ELISA tests of MIG further confirmed the Luminex results. Consequently, we conducted further analysis to explore the correlation between these cytokine levels and classic CSF parameters. We discovered a strong negative correlation between CSF MIG and CSF/blood glucose ratio in TBM infection, as well as positive correlations between CSF FGF.basic, G-CSF, MCP-3, MIP-1α, TNF-α, and CSF leukocyte counts or protein levels. Furthermore, we observed that CSF MIG exhibited the highest AUC value in TBM, with a value of 0.92 (95% CI 0.82–1.00).

Th1 cell-mediated immunity has been extensively implicated in the pathogenesis of TB and serves as a significant source of TB biomarkers (24). CXC chemokine receptor 3 ligands, also known as IFN-γ-inducible chemokines, play a critical role in countering Mtb infection by specifically targeting the Th1 pathway (25). Among these ligands, MIG (CXCL9) is of particular interest, along with IP-10 (CXCL10) and IFN-inducible T cell α chemoattractant (CXCL11). MIG secretion primarily relies on IFN-γ during infection and is believed to be essential for granuloma formation and subsequent host defense against *M. tuberculosis* (26). Previous studies have identified MIG as a potential diagnostic tool for TB infection (9, 27, 28). A systematic review and meta-analysis evaluating the predictive accuracy of MIG found pooled sensitivity and specificity both to be 84% among patients with culture-positive TB (29). Moreover, a study demonstrated that MIG outperformed IFN-γ in diagnosing active TB based on ROC analysis (27). The areas under the curve (95% CI) for differentiating active pulmonary TB from other groups were 0.893 (0.864–0.924) for IFN-γ and 0.944 (0.922–0.965) for MIG, with corresponding sensitivities and specificities of 84.9% and 79.8% for IFN-γ and 92.5% and 85.2% for MIG, respectively. In our present study, we identified MIG as a promising biomarker for further evaluating its diagnostic value in TBM infection. We determined the optimal cutoff value to be 783.30 pg/mL in CSF for distinguishing TBM infection from controls. Additionally, we observed negative correlations between CSF MIG levels and the CSF-to-blood glucose ratio in TBM infections. These findings align with previous studies that demonstrated negative correlations between CSF IL-6 levels and the CSF-to-blood glucose ratio (*r* = −0.4991, *P* = 0.0009) and suggested IL-6 as a promising biomarker for CNS infection (18). The concentration of CSF glucose is influenced by the anaerobic metabolic activity of bacteria or fungi and is closely associated with systemic glucose levels, which can be affected by various factors (30).

IL-18, initially isolated from the serum of *Mycobacterium bovis* BCG-infected mice and described as “IFN-γ-factor” in 1989 (31), was found to induce IFN-γ production and plays a significant role in promoting Th1 responses in TBM (32 – 35). Some studies have suggested that IL-18 might be applicable in designing novel diagnostic tests for TB (36 – 39). In our study, only IL-18 exhibited significantly higher levels in the CSF and serum of TBM patients than in the control groups, and significant differences were observed in its CSF levels after treatment. We also demonstrated that IL-18 showed relatively high predictive efficiency, with an AUC of 0.83 (95% CI 0.67–0.98) and a sensitivity of 0.85, ranking it first among the analyzed cytokines. When combined with MIG, the prediction model exhibited higher sensitivity (0.92), indicating excellent predictive value.

Despite the promise demonstrated by the aforementioned CSF host inflammatory biomarkers (FGF.basic, G-CSF, MCP-3, MIP-1α, TNF-α), the process of acquiring CSF through lumbar puncture may present challenges in resource-limited settings when performing CSF-based tests. In light of our correlation analyses and AUC results, our research suggests that MIG could serve as a highly specific serum biomarker for diagnosing TBM with promising accuracy. The advantage of utilizing blood-based biomarkers lies in their potential for detection through fingerprick blood samples, providing a convenient means for monitoring TBM treatment response.

Several limitations should be noted in our study. First, the sample size in each group, particularly the non-infection cohort, was insufficient, and further assessment of the potential biomarkers identified in this preliminary study is necessary in larger participant numbers. We restricted the total number of patients in this study to ensure that they could be accommodated on one plate for CSF and serum samples, along with the standard curve, to avoid inter-plate variation. Future studies should include TBM patients with HIV infection and validate the promising biomarkers using separate training and testing sets of samples. Additionally, the CM group in our study may not provide a comprehensive representation. In clinical practice, meningitis encompasses a broader range of causative factors, including viral and bacterial infections as well as autoimmune disorders, which were not included in this study. Moreover, the direct effects of *M. tuberculosis* on pathogenesis were not explored. We could only detect the bacterial load as positive or negative in our lab, so it is difficult to determine the correlation between cytokine levels and bacterial load. Further studies on the relationships between the loads of microbes and concentrations of cytokines are needed. Finally, some TBM patients were diagnosed based on clinical findings without definitive pathogenic evidence. The possibility of misclassification exists despite our efforts.

In conclusion, we have identified MIG as a specific marker for diagnosing TBM in CSF or serum. However, our preliminary findings require confirmation in larger studies. If validated, these findings have the potential to facilitate the timely diagnosis of TBM and improve patient outcomes.

## MATERIALS AND METHODS

### Study cohort

For this retrospective study, a total of 232 patients with suspected CNS infection who were admitted to the First Affiliated Hospital of Fujian Medical University between January 2021 and March 2023 were included. The definition of suspected CNS infections was based on the diagnostic criteria of meningitis, encephalitis, meningoencephalitis, and meningomyelitis as a previous study reported, such as fever, headache, vomiting, altered consciousness, seizures, a new onset of focal neurologic findings, and signs of meningeal irritation (40, 41). The exclusion criteria were as follows: a diagnosis of autoimmune encephalitis, bacterial meningitis/encephalitis, viral meningitis/encephalitis; refusal to undergo lumbar puncture; or any contraindication for such puncture. Among the 232 patients, 33 eligible individuals were selected for further cytokine studies and divided into three groups according to their final diagnoses: TBM (*n* = 17), CM (*n* = 10), and NF (*n* = 6). Posttreatment CSF samples from 11 TBM patients were available for cytokine estimation.

The inclusion criteria of TBM patients were as follows (42): (i) symptoms and signs of meningitis, including one or more of the following: headache, vomiting, fever, neck stiffness, convulsions, focal neurological deficits, altered consciousness, or lethargy; (ii) meeting at least one of the following subconditions: (i) CSF: cells 10–500 per μL, lymphocytic predominance (>50%), protein concentration >1 g/L, CSF to serum glucose ratio of <50% or an absolute CSF glucose concentration of <2.2 mmol/L; (ii) cerebral imaging criteria: hydrocephalus, basal meningeal enhancement, tuberculoma, infarct, or precontrast basal hyperdensity; (iii) evidence of tuberculosis elsewhere. Patients were then classified into definite, probable, possible, or not tuberculous meningitis according to these diagnostic criteria as reported (42). A definitive diagnosis of TBM was established based on positive culture results for Mtb, visualization of acid-fast bacilli (AFB), or evidence from commercial nucleic acid amplification tests for Mtb, including Xpert MTB/RIF and metagenomic next-generation sequencing (mNGS), in the CSF. The diagnosis of probable or possible TBM was made using a diagnostic scoring system (42). Probable TBM was defined as a diagnostic score of 12 or higher with available imaging or a score of 10 or higher without imaging. For possible TBM, a diagnostic score of 6–11 was required with available imaging or a score of 6–9 without imaging (42). CM was confirmed when CSF samples tested positive for India ink staining, fungal culture, mNGS, or CrAg. Additionally, six patients who had a CSF leukocyte count <5 × 10^6^/L and were excluded from CNS infection were included in the NF group. The serum NF group tested by ELISA also included another seven patients [aged 30–50 years (mean: 40.5); two were male] without CNS infection.

The remaining CSF and serum samples were used for further cytokine tests from the three groups of patients using the following exclusion criteria: (i) the volume of CSF or serum was not enough for cytokine detection; (ii) the quality of CSF was too low to perform cytokine detection, such as hemolytic CSF. All patients tested negative for HIV in their serum. Routine tests included CSF cultures, AFB staining, Indian ink staining, and blood cultures in all cases. Detailed clinical information, including age, gender, clinical symptoms and signs, CSF characteristics, and imaging findings, was collected for all groups.

### Specimen handling and routine CSF testing

A lumbar puncture was performed using an atraumatic needle, and 10 to 20 mL of CSF was collected. Simultaneously, 2 mL of whole blood was drawn for serum cytokine analysis. The collected CSF and serum samples were centrifuged (5,500 × *g*, 10 minutes, 4°C), and the resulting supernatant was divided into 500 µL aliquots in polypropylene tubes. The aliquots were then stored at −80°C for 120 minutes. Routine CSF work-up included biochemical and pathological studies, such as cytology, biochemistry, bacterial or fungal cultures, and smears, which were conducted at the Laboratory Medicine Center of the First Affiliated Hospital of Fujian Medical University. Additionally, cytokine analysis was performed on 11 CSF samples collected approximately 1 month after the initiation of anti-tuberculous therapy.

### Measurement of cytokines

The supernatant of serum samples was diluted fourfold. A 50 µL sample of the diluted serum and an undiluted 50 µL sample of CSF supernatant were taken for testing. The levels of 48 cytokines, including cutaneous T cell attracting chemokine, eosinophil chemotactic protein, FGF.basic, G-CSF, GM-CSF, growth-related gene-α, HGF, IFN-α2, IFN-γ, IL-1α, IL-1β, IL-1ra, IL-2, IL-2Rα, IL-3, IL-4, IL-5, IL-6, IL-7, IL-8, IL-9, IL-10, IL-12(P40), IL-12(P70), IL-13, IL-15, IL-16, IL-17, IL-18, IP-10, LIF, MCP-1, MCP-3, M-CSF, MIF, MIG, MIP-1α, MIP-1β, β-NGF, platelet-derived growth factor (PDGF)-BB, regulated upon activation normal T cell expressed and secreted factor, SCF-1α, SCF-β, stromal cell-derived factor-1α, TNF-α, TNF-β, TRAIL, and VEGF, were simultaneously measured using the Bio-Plex Pro Human Cytokine Screening 48-plex Panel (#12007283; Bio-Rad Laboratories, Hercules, CA, USA), following the manufacturer’s instructions as previously described (43). This panel comprised biologically significant adaptive immunity cytokines, proinflammatory cytokines, and anti-inflammatory cytokines. To ensure optimal comparability within each compartment, all CSF and serum samples were measured on a single multiplex plate. After detection using the Luminex 200 detector (Luminex Corporation, Austin, TX, USA), the obtained fluorescence signals were automatically calculated by the software to generate an output file in Excel format. All results were expressed in pg/mL.

### MIG analysis by ELISA

CSF and serum levels of MIG were detected by the Quantikine ELISA Human MIG Immunoassay Kit (catalog number: DCX900; R&D Systems Europe Ltd., Abingdon, England) according to the manufacturer’s instructions. Samples were not diluted before analysis except for TBM patients CSF due to the high concentration indicated by the Luminex assay. The Quantikine ELISA for Human MIG had a detection limit of 3.84 pg/mL and a measuring range of 31.3–2,000 pg/mL. The manufacturer of the assay kits refers to the intra-assay coefficient of variation (%), which was indicated at 3.1% to 3.9%.

### Statistical analysis

Continuous variables were presented as medians (interquartile ranges), while categorical variables were expressed as numbers (%). Student’s *t*-test was used to compare continuous variables that followed a normal distribution, while non-parametric tests (Mann-Whitney *U* test) were used for continuous variables that did not meet the normality assumption. However, in a few samples, the measured concentrations were below the detection limit and were excluded from further analysis. The correlation clustering heatmap between cytokine levels was displayed using the corrplot package (version 0.92) of R, in which the correlation coefficient was calculated using the Pearson method. Associations of the cytokine levels with TBM, CM, and NF were analyzed by PCA, which was performed using the CSF/serum levels of the 48 cytokines measured by Luminex. Each dot represented a sample projected in the two main principal components (PC1 and PC2), and the dots were colored according to the cohort they belonged to. The ggord package (version 1.1.7) of R was used for PCA analysis. The Spearman’s rank correlation test was performed to assess the associations between CSF cytokines and CSF parameters, including leukocytes, proteins, and the CSF/blood glucose ratio. The laboratory personnel were blinded to the clinical information associated with the samples. Statistical significance was set at *P* < 0.05 (two-tailed). The performance of the diagnostic models was evaluated using ROC curve analysis. Sensitivity, specificity, and accuracy, along with their corresponding 95% CI, were calculated. Data analysis was conducted using R (version 4.05) and GraphPad version 9 (GraphPad Software, San Diego, California, USA).

## SUPPLEMENTAL MATERIAL

Supplemental material spectrum.02246-23-s0001.docx Fig. S1 to S3; Tables S1 and S2.

ASM does not own the copyrights to Supplemental Material that may be linked to, or accessed through, an article. The authors have granted ASM a non-exclusive, world-wide license to publish the Supplemental Material files. Please contact the corresponding author directly for reuse.
