# Supplementary material for: Systemic and cerebrospinal fluid biomarkers for tuberculous meningitis identification and treatment monitoring
Source: Microbiol Spectr. 2023 Dec 4;12(1):e02246-23. doi: 10.1128/spectrum.02246-23 (PMC10783035; doi:10.1128/spectrum.02246-23)
Supplement: Supplemental material — Fig. S1 to S3; Tables S1 and S2. [file spectrum.02246-23-s0001.docx]

**Supplementary Data**

**Systemic and cerebrospinal fluid biomarkers for tuberculous meningitis identification and treatment monitoring**

**Xiang-Ping Yao^1,2#^, Jian-Chen Hong^3#^, Zai-Jie Jiang^1#^,** **Yu-Ying Pan^1^, Xiao-Feng Liu^4^, Jun-Mei Wang^1^, Rui-Jie Fan^1^, Bi-Hui Yang^1^, Wei-Qing Zhang^4^, Qi-Chao Fan^5^, Li-Xiu Li^6^, Bi-Wei Lin^1^*, Miao Zhao^1,2^****

^1^Department of Neurology and Institute of Neurology of First Affiliated Hospital, Institute of Neuroscience, and Fujian Key Laboratory of Molecular Neurology, Fujian Medical University, Fuzhou 350005, China

^2^Department of Neurology, National Regional Medical Center, Binhai Campus of the First Affiliated Hospital, Fujian Medical University, Fuzhou 350212, China

^3^Department of Gastrointestinal Surgery, the First Affiliated Hospital, Fujian Medical University, Fuzhou 350005, China

^4^Department of Laboratory Medicine, the First Affiliated Hospital, Fujian Medical University, Fuzhou 350005, China

^5^Department of Infectious Disease, the First Affiliated Hospital, Fujian Medical University, Fuzhou 350005, China

^6^Department of Oncology, Fuzhou Pulmonary Hospital of Fujian, Fuzhou 350005, China

**^#^** These authors contributed equally

*Corresponding Author. Department of Neurology and Institute of Neurology of First Affiliated Hospital, Institute of Neuroscience, and Fujian Key Laboratory of Molecular Neurology, Fujian Medical University, Fuzhou 350005, China. E-mail address: linbiwei@fjmu.edu.cn

**Corresponding Author. Department of Neurology and Institute of Neurology of First Affiliated Hospital, Institute of Neuroscience, and Fujian Key Laboratory of Molecular Neurology, Fujian Medical University, Fuzhou 350005, China. E-mail address: zhaomiao91@fjmu.edu.cn


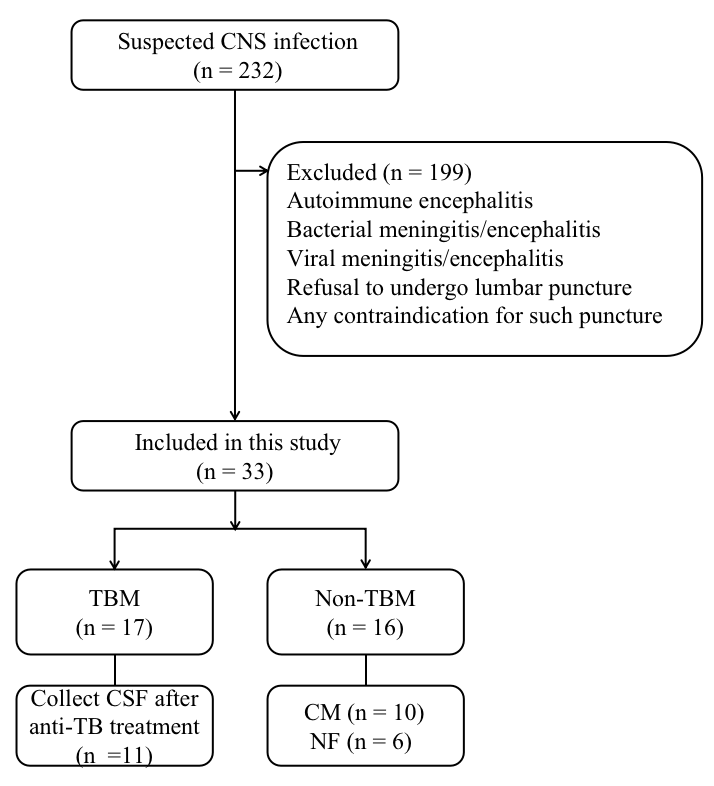


**FIG S1** A flowchart showing the study design and classification of study participants. A total of 232 patients with suspected CNS infection were enrolled. Of these patients, 17 were diagnosed with TBM. Among them, posttreatment CSF samples from 11 TBM patients were available for cytokine estimation. The non-TBM group included CM (n = 10) and NF (n = 6). CNS, central nervous system; TBM, tuberculous meningitis; Non-TBM, individuals presenting with symptoms and investigated for TB but TBM ruled out. CSF, cerebrospinal fluid; CM, cryptococcal meningitis; NF, non-infection.


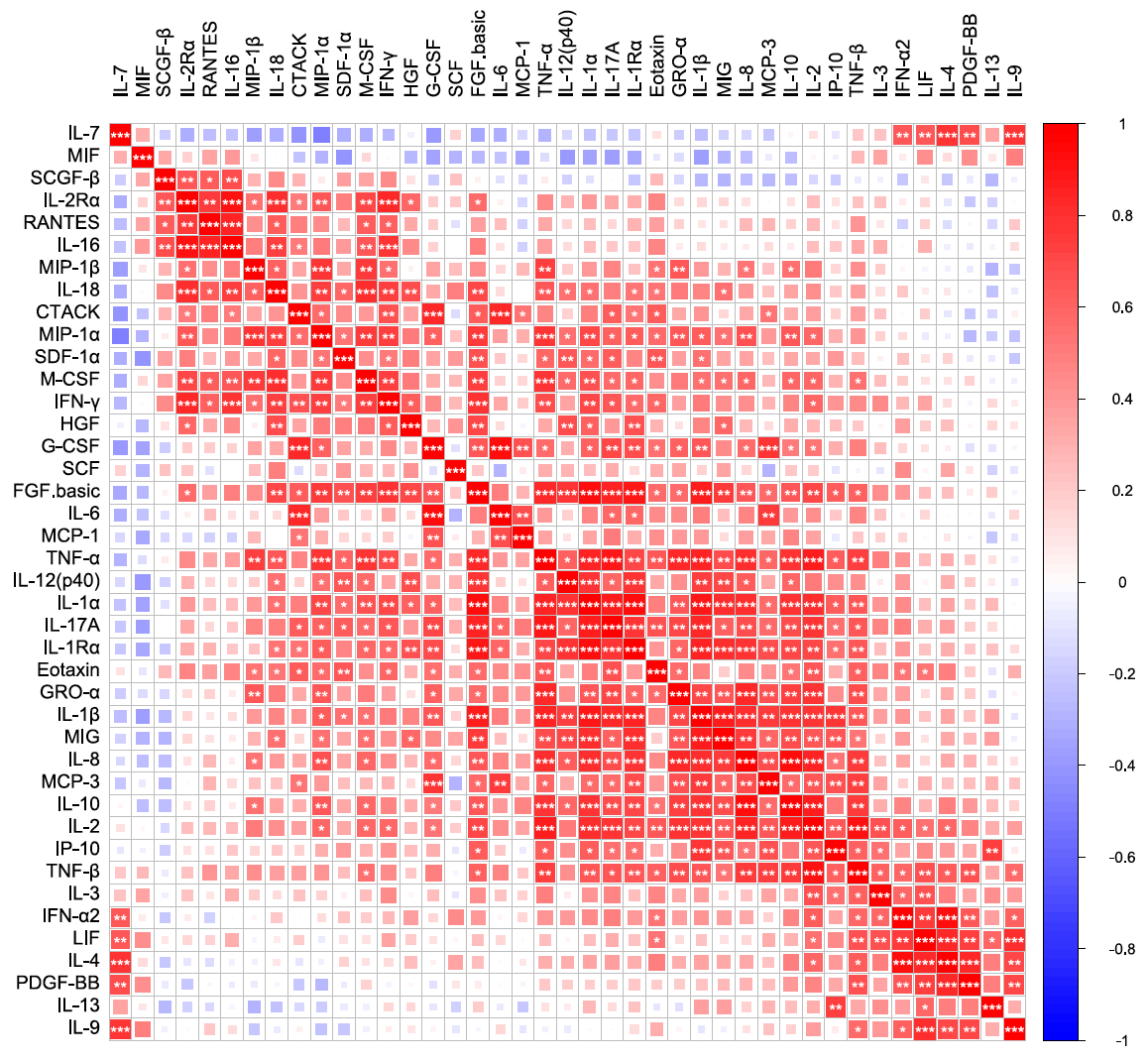


**FIG S2** Clustering of correlations between each CSF cytokine level in TBM patients. Color code indicates R values of correlations calculated using Pearson’s correlation coefficient. CSF: cerebrospinal fluid; TBM, tuberculous meningitis.


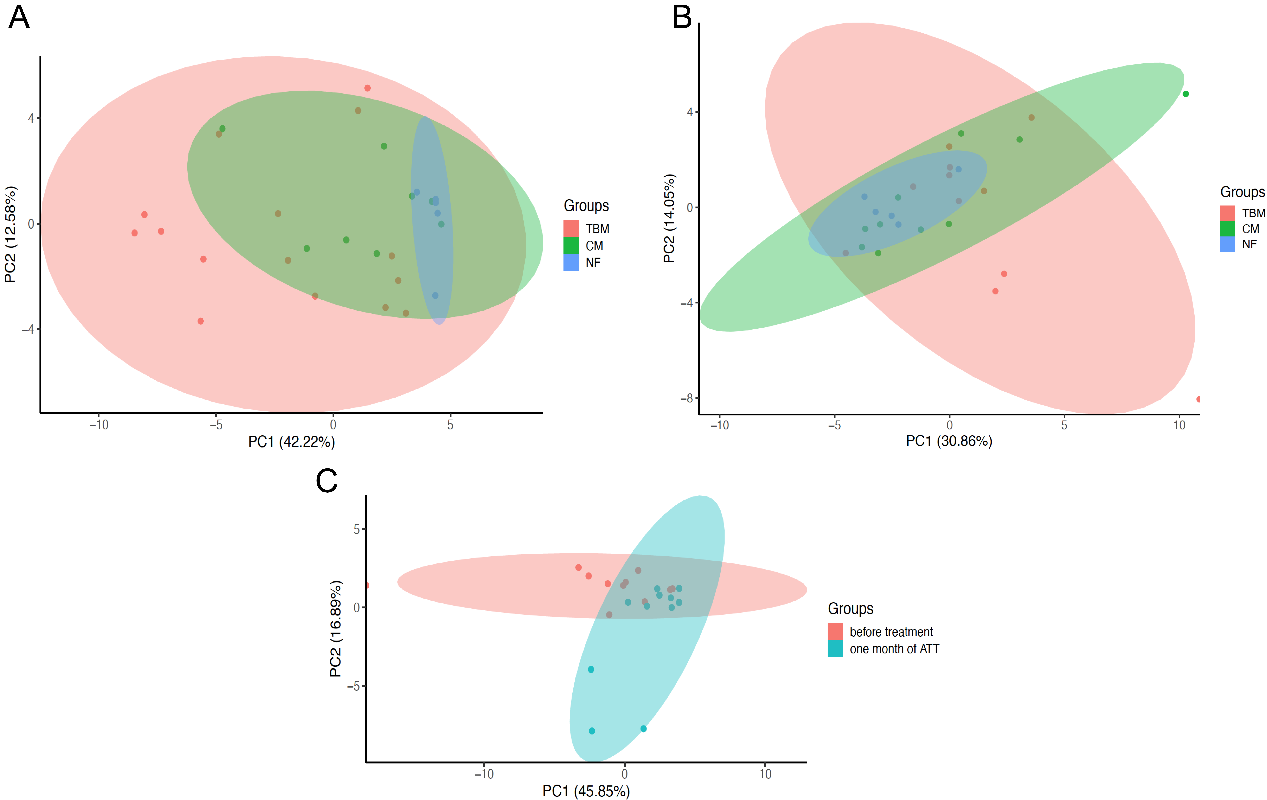


**FIG S3** Principal component analysis based on cytokines in CSF/serum of patients with TBM, CM, and NF. Clear separations of the three groups (TBM, CM, and NF) were observed in CSF (A) and serum (B). Visual clustering of TBM cases were seen in before and one month after ATT (C). Each dot represents 1 participant based on the values of all cytokines studied. CSF: cerebrospinal fluid; TBM, tuberculous meningitis; CM, cryptococcal meningitis; NF, non-infection; ATT, anti-tuberculosis treatment.

**Table S1** The detail changes in CSF profiles of the 11 TBM patients after one months of anti-tuberculous therapy.

|  | Pretreatment | | | | | Posttreatment | | | | |
| --- | --- | --- | --- | --- | --- | --- | --- | --- | --- | --- |
| No. | P  (mmH2O) | WBC  (/μL) | Glucose  (mmol/L) | Protein  (g/L) | CSF/BS  glucose ratio (%) (%) | P  (mmH2O) | WBC  (/μL) | Glucose  (mmol/L) | Protein  (g/L) | CSF/BS  glucose ratio (%) |
| 2 | 300 | 396 | 1.70 | 2.28 | 28.43 | 130 | 82 | 2.17 | 1.10 | 34.01 |
| 3 | NA | 209 | 2.53 | 2.20 | 31.39 | 150 | 73 | 3.50 | 0.56 | 31.62 |
| 4 | 130 | 1011 | 2.40 | 3.20 | 42.25 | 130 | 101 | 3.37 | 0.35 | 55.98 |
| 6 | 220 | 53 | 1.73 | 1.72 | 26.82 | 210 | 20 | 1.69 | 0.46 | NA |
| 7 | 160 | 109 | 1.53 | 3.11 | 26.38 | 140 | 42 | 2.22 | 1.05 | 27.85 |
| 8 | 180 | 355 | 2.21 | 0.78 | 37.84 | 155 | 40 | 2.21 | 0.77 | NA |
| 9 | 170 | 74 | 2.85 | 3.63 | NA | 130 | 5 | 3.14 | 1.43 | 65.55 |
| 10 | NA | 38 | 2.26 | 1.69 | NA | NA | 14 | 3.38 | 0.93 | NA |
| 11 | 75 | 104 | 3.18 | 0.62 | 27.85 | 130 | 104 | 4.63 | 0.26 | 33.72 |
| 12 | 60 | 176 | 2.21 | 5.16 | 34.32 | 150 | 60 | 2.30 | 4.23 | 30.83 |
| 17 | 235 | 62 | 4.55 | 0.32 | NA | 280 | 24 | 7.13 | 0.55 | 46.09 |

Abbreviation: No., case number; P, intracranial pressure; WBC, white blood cell; CSF, cerebrospinal fluid; BS, blood serum; NA, not available.

**Table S2** MIG concentrations in the CSF and serum of TBM patients as compared to the control group.

| Parameter | TBM | CM | NF | *p*-value |
| --- | --- | --- | --- | --- |
| CSF MIG | 7609.57  (968.06-19800.29)  N = 17 | 422.74  (212.28-1362.27)  N = 10 | 77.26  (48.82-122.38)  N = 6 | TBM vs. CM, *p* = 0.0030  TBM vs. NF, *p* = 0.0021 |
| Serum MIG | 384.79  (287.09-780.55)  N = 12 | 306.91  (202.83-368.32)  N = 10 | 185.73  (129.45-258.84)  N = 13 | TBM vs. CM, *p* = 0.0843  TBM vs. NF, *p* = 0.0202 |

The data were presented as median (interquartile range), two groups were compared using Student’s t-test. patients. Concentrations in pg/mL of MIG were showed. TBM, Tuberculous meningitis; CM, cryptococcal meningitis, NF: non-infection.
